# Multiple steroid and thyroid hormones detected in baleen from eight whale species

**DOI:** 10.1093/conphys/cox061

**Published:** 2017-11-09

**Authors:** Kathleen E Hunt, Nadine S Lysiak, Jooke Robbins, Michael J Moore, Rosemary E Seton, Leigh Torres, C Loren Buck

**Affiliations:** 1 Biological Sciences & Center for Bioengineering Innovation, Northern Arizona University, Flagstaff, AZ, USA; 2 University of Massachusetts-Boston, Boston, MA, USA; 3 Center for Coastal Studies, Provincetown, MA, USA; 4 Biology Department, Woods Hole Oceanographic Institution, Woods Hole, MA, USA; 5 Allied Whale, College of the Atlantic, Bar Harbor, ME, USA; 6 Department of Fisheries and Wildlife, Oregon State University, Newport, OR, USA

**Keywords:** Baleen, cetaceans, hormones, marine mammals, reproduction, stress

## Abstract

Recent studies have demonstrated that some hormones are present in baleen powder from bowhead (*Balaena mysticetus)* and North Atlantic right (*Eubalaena glacialis*) whales. To test the potential generalizability of this technique for studies of stress and reproduction in large whales, we sought to determine whether all major classes of steroid and thyroid hormones are detectable in baleen, and whether these hormones are detectable in other mysticetes. Powdered baleen samples were recovered from single specimens of North Atlantic right, bowhead, blue (*Balaenoptera* [*B*.]*musculus*), sei (*B. borealis*), minke (*B. acutorostrata*), fin (*B. physalu*s), humpback (*Megaptera novaeangliae*) and gray (*Eschrichtius robustus*) whales. Hormones were extracted with a methanol vortex method, after which we tested all species with commercial enzyme immunoassays (EIAs, Arbor Assays) for progesterone, testosterone, 17β-estradiol, cortisol, corticosterone, aldosterone, thyroxine and tri-iodothyronine, representing a wide array of steroid and thyroid hormones of interest for whale physiology research. In total, 64 parallelism tests (8 species × 8 hormones) were evaluated to verify good binding affinity of the assay antibodies to hormones in baleen. We also tested assay accuracy, although available sample volume limited this test to progesterone, testosterone and cortisol. All tested hormones were detectable in baleen powder of all species, and all assays passed parallelism and accuracy tests. Although only single individuals were tested, the consistent detectability of all hormones in all species indicates that baleen hormone analysis is likely applicable to a broad range of mysticetes, and that the EIA kits tested here perform well with baleen extract. Quantification of hormones in baleen may be a suitable technique with which to explore questions that have historically been difficult to address in large whales, including pregnancy and inter-calving interval, age of sexual maturation, timing and duration of seasonal reproductive cycles, adrenal physiology and metabolic rate.

## Teaser

Progesterone, testosterone, 17β-estradiol, cortisol, corticosterone, aldosterone, thyroxine and tri-iodothyronine are detectable in baleen of eight whale species. The presence of a broad array of hormones in baleen of multiple species indicates that baleen hormone analysis may be widely applicable for studies of reproduction, stress and metabolism of the large whales.

## Introduction

Large whales are of interest to conservation biologists due to their key ecological role, unique biological traits (e.g. large body size, long lifespan) and continued low population numbers relative to historic norms (Magera *et al.*, 2013; Atkinson *et al.*, 2015; Thomas and Reeves, 2015). Despite the cessation of commercial whaling, many whale populations remain threatened or endangered, and most are subject to a wide variety of anthropogenic impacts (Magera *et al.*, 2013; Thomas and Reeves, 2015). Unfortunately, management and recovery efforts have been hampered by the fact that many basic aspects of cetacean life histories remain poorly understood, largely due to the logistic complications of locating and sampling free-swimming whales at sea (Hunt *et al.*, 2013; Atkinson *et al.*, 2015). Perhaps most problematic from a physiological perspective, there is still no proven method for live-capture of large whales, nor for collection of blood samples from free-swimming whales. Without traditional physiological analyses of plasma samples, many physiological features of the large whales still remain unknown or only roughly estimated, including such essential information as gestation length, inter-calving interval, reproductive rate, reproductive cycling (e.g. potential seasonal estrous cycles in females and/or seasonal testosterone cycles in males), and physiological responses to environmental stressors (both anthropogenic and natural).

Endocrinological analysis of alternative (non-plasma) sample types offers a potential avenue forward. In terrestrial taxa, conservation biologists now routinely employ endocrine techniques for non-plasma sample types that are more easily garnered than blood, including such sample types as faeces, hair and feather (Schwarzenberger, 2007; Sheriff *et al.* 2011; Terwissen *et al.*, 2014; Dettmer *et al.*, 2015; Romero and Fairhurst, 2016). Such approaches often focus on the steroid hormones and to a lesser degree the thyroid hormones; steroid and thyroid hormones are highly conserved across vertebrates (i.e. unlike many peptide hormones; Miller, 2005; Kawauchi and Sower, 2006), do not degrade rapidly, and are known to be deposited in a wide assortment of body tissues (Bentley, 1998; Amaral, 2010). This suite of hormones is also ideally suited for addressing questions of conservation interest. The gonadal steroids play key roles in reproduction (progestagens and estrogens for females, and androgens for males), while the adrenal steroids are critical for coordination of the vertebrate response to stress (particularly the glucocorticoids cortisol and corticosterone) as well as osmotic regulation (e.g. the mineralocorticoid aldosterone) (Bentley, 1998). Thyroid hormones, generally thought to be drivers of metabolic rate in the mammals (Hulbert, 2000), have received less attention from conservation biologists than the steroids, but are proving to be increasingly useful for wildlife physiology studies as indices of nutritional state and activity (Eales, 1988; Ayres *et al.*, 2012; Joly *et al.*, 2015; Wilsterman *et al.*, 2015). Thyroid hormone studies on wildlife usually focus either on tri-iodothyronine (T3), the active hormone in tissues, and/or its immediate precursor thyroxine (T4), the major circulating pro-hormone (Bentley, 1998).

Significant progress has been made at recovering and quantifying some of the hormones described above from alternative tissue types in cetaceans, particularly faecal samples, respiratory vapour (‘blow’), and biopsy dart samples of skin and blubber (Rolland *et al.*, 2005; Hunt *et al.*, 2006, 2014a; Kellar *et al.*, 2006; reviewed in Hunt *et al.*, 2013; De Mello and de Oliveira, 2016) and most recently earwax plugs (Trumble *et al.*, 2013). For example, progestagens in faeces, blow and blubber are significantly elevated in pregnant females, while faecal glucocorticoids and mineralocorticoids have been shown to correlate with exposure to environmental stressors such as chronic ocean noise and fishing gear entanglement (Rolland *et al.*, 2005; Hunt *et al.*, 2006; Hogg *et al.*, 2009; Kellar *et al.*, 2013; Burgess *et al.*, 2017). However, despite these encouraging advances, sample collection rate is low for all of these sample types. Especially, it is rare to obtain repeated samples from the same individual whale over time (Hunt *et al.*, 2013). Without repeated sampling from individuals across time, it remains difficult to study physiological aspects of the long reproductive cycles of cetaceans as well as long-term phenomena such as responses to chronic stress.

Baleen has recently been recognized as a tissue type that may contain a longitudinal time series of endocrine history covering multiple years (Hunt *et al.*, 2014b, 2016). In mysticete whales, vertical strips or ‘plates’ of baleen are suspended in parallel along the right and left sides of the whale’s mouth, serving as a filter-feeding apparatus (St. Aubin *et al.*, 1984). Stable isotope (SI) data indicate that each baleen plate grows continuously and slowly from a highly vascularized root region in the upper jaw, wearing away steadily at the distal (lower) tip (Mitani *et al.*, 2006; Lubetkin *et al.*, 2008, 2012; Lysiak *et al.*, 2008; Ryan *et al.*, 2013). The number of years of baleen growth represented by a given baleen plate can often be determined with high accuracy via counting of annual cycles in SI ratios along the length of the baleen plate, since SI ratios often change seasonally in baleen as these migratory species alternate between summer prey and winter prey (Mitani *et al.*, 2006; Lubetkin *et al.*, 2008, 2012; Lysiak *et al.*, 2008; Ryan *et al.*, 2013). Our recent studies using these techniques (hormonal analyses combined with SI analyses) have demonstrated that progesterone and cortisol are detectable in baleen of bowhead whales (*Balaena mysticetus*, ‘bowhead’) and North Atlantic right whales (*Eubalaena glacialis*, ‘right’), and that peaks in both hormones occur in regions of baleen known (from the SI data and resulting baleen growth rate estimates) to have grown during documented pregnancies (Hunt *et al.*, 2014b, 2016). Thus, baleen quite likely contains a longitudinal endocrine history that spans the time period of growth of the baleen plate, which can be a decade or more for the species with longest baleen, bowheads and rights. Even in species with shorter baleen, a single baleen plate encompasses at least a full year of growth, ~1.3–1.4 years in the gray whale (*Eschrichtius robustus*, ‘gray’; Caraveo-Patiño *et al.*, 2007) and minke whale (*Balaenoptera acutorostrata*, ‘minke’; Mitani *et al.*, 2006) and usually multiple years in the humpback whale (*Megaptera novaeangliae*, ‘humpback’); finback whale (*B. physalus*, ‘fin’) and sei whale (*B. borealis*, ‘sei’; Bentaleb *et al.*, 2011; Ryan *et al.*, 2013; Eisenmann *et al.*, 2016) (no estimates are yet available for blue whale, *B. musculus*, ‘blue’). These growth durations are sufficiently long to encompass complete pregnancies, inter-calving intervals in most species, and potentially long-term responses to a variety of environmental stressors, data that would otherwise require collection of many dozens of samples. For example, a single baleen plate from an adult right whale contains a continuous time series of endocrine information that spans 9–10 years (Hunt *et al.*, 2016, 2017), perhaps comparable to a collection of weekly or monthly plasma samples collected repeatedly from the same individual over an entire decade. Two additional advantages of baleen are, first, that it is a dry and long-lasting tissue that holds steroid hormones in stable condition for at least one decade (Hunt *et al.*, 2016); second, considerable archives of baleen samples exist in stranding archives and in natural history museums that may enable retrospective study of historic populations.

Hormonal analysis of baleen therefore has the potential to reveal both annual and multi-year patterns in physiology from both modern and historic specimens. However, to date only two hormones have been investigated (progesterone and cortisol; Hunt *et al.*, 2014b, 2016) and in only two species, right and bowhead, which are closely related (Marx and Fordyce, 2015). It is unknown whether hormones are also present in baleen of other cetacean taxa, and it is also unclear whether other hormones may be present as well—particularly testosterone, 17β-estradiol, corticosterone, aldosterone and thyroid hormones—and, specifically, whether they might be detectable with commercially available immunoassay kits (the most widespread and lowest-cost endocrine technique presently available). If any of these hormones prove detectable with immunoassays, at least two types of validations will be necessary before the technique can enter widespread use (Palme, 2005; Goymann, 2012; Kersey and Dehnhard, 2014). First, laboratory validations (the focus of this study) must be performed to verify that the assays can accurately quantify the desired hormones, i.e. even in the presence of baleen extract. Second, physiological validations (also known as ‘biological validations’) will also be necessary for each hormone and each species, to verify that baleen hormone concentrations do in fact correspond to physiological state of the animal (e.g. demonstration that baleen of known-pregnant whales contains elevated progesterone and elevated glucocorticoids, as shown for bowheads and rights in Hunt *et al.*, 2014b, 2016, 2017). Note that a third class of validations, pharmacological validations (infusions of hormones such as ACTH, hormone receptor blockers, radiolabelled hormones, etc.), are typically not possible in large whales (Hunt *et al.*, 2013) but, fortunately, thorough attention to laboratory validations and physiological validations can demonstrate whether the assay in question can accurately identify animals of the desired physiological state.

As a first step in investigating whether baleen hormone technique is of widespread applicability to baleen whales, we performed laboratory validations for eight hormones in baleen (physiological validations are being addressed in parallel studies, to be published separately). Initial laboratory validations of immunoassays for any novel sample type from a given species commonly include a parallelism test (assay of serially diluted sample alongside hormone standards) and an accuracy test (assay of a set of hormone standards that have been spiked with pooled sample), both typically performed initially on a single specimen from the species in question (Grotjan and Keel, 1996). A parallelism test confirms that the assay antibody exhibits good binding affinity to the desired hormone in that sample type from that species; the accuracy test verifies that the assay is able to distinguish high from low concentrations with acceptable mathematical accuracy (Grotjan and Keel, 1996). Therefore, as a first step in investigating whether baleen hormone technique is of widespread applicability to baleen whales, we tested baleen of eight mysticete species (right, bowhead, blue, sei, minke, fin, humpback and gray), representing a broad range of mysticete taxa, for parallelism in commercial enzyme immunoassay (EIA) kits for progesterone, testosterone, 17β-estradiol, cortisol, corticosterone, aldosterone and the thyroid hormones T3 and T4, representing all major classes of vertebrate steroid and thyroid hormones. Where sample volume permitted, we tested accuracy as well. Our investigation includes all known genera of mysticete whales except *Caperea*, the pygmy right whale (Marx and Fordyce, 2015). Our goals were to determine (i) which species of mysticetes have detectable hormones in baleen, (ii) which (if any) of the eight hormones are detectable and (iii) whether commercial EIAs perform well with baleen extract (i.e. good parallelism and accuracy).

## Methods

### Baleen samples

Since this study required semi-destructive sampling of rare specimens, and initial parallelism validations can be accomplished with single individuals, sample sizes in this pilot trial were restricted to one individual per species and one baleen plate per individual. One baleen plate each from single specimens of right, bowhead, blue, sei, minke, fin, humpback and gray whales were selected for study from baleen archived in previous years by US marine mammal stranding networks on the east and west coasts (Table 1). For blue, fin and gray whales, a baleen plate from a second individual whale was later studied to further inspect parallelism results for cortisol; in these cases the individuals are distinguished as #1 and #2, e.g. blue whale #1 and blue whale #2. All plates had been stored at room temperature for multiple years, in some cases decades, between date of specimen collection and hormone assays in 2016–17 (Table 1). Since assay validations require pulverization of a relatively large area of baleen (i.e. in order to provide ample extract for testing), less-valuable specimens were selected for these initial validations; therefore, several specimens do not have associated necropsy data and some are of unknown sex and unknown age-at-death (Table 1). Note that sex of the whale, age at death, reproductive state, cause of death, years in storage of the baleen, and potentially storage conditions may all affect hormone concentrations in baleen (see, e.g. Hunt *et al.*, 2014b, 2016, 2017), but this was not a concern in this study given that our primary focus was simply whether hormones are present and detectable.

**Table 1:** Baleen specimens used for testing hormone assay parallelism and accuracy

Species	Short id	Carcass accession code	Holding institution^a^	Sex	Collection year	Other information
*Eubalaena glacialis*	Right	VMSM2004-1004	WHOI	Female	2004	Eg #1004 ‘Stumpy,’ adult female, died due to shipstrike
*Balaena mysticetus*	Bowhead	n/a	NAU	Unk	Before 1991	Collected in legal native subsistence hunt; historic educational specimen
*Balaenoptera musculus*	Blue #1	MMASYBM9812	NBWM/WHOI	Male	1998	‘KOBO,’ subadult, 2002cm length, died due to shipstrike; museum display specimen
*Balaenoptera musculus*	Blue #2	HMSC151101Bm	OSU	Male	2015	Necropsy specimen; adult, 2127 cm length
*Balaenoptera borealis*	Sei	COA150609Bb	COA	Female	2015	Necropsy specimen from marine mammal stranding network
*Balaenoptera acutorostrata*	Minke	COA140717Ba	COA	Female	2014	Necropsy specimen from marine mammal stranding network
*Balaenoptera physalus*	Fin #1	COA830916Bp	COA	Unk	1983	Necropsy specimen from marine mammal stranding network
*Balaenoptera physalus*	Fin #2	HMSC090306Bp	OSU	Male	2009	Subadult, 1678 cm length
*Megaptera novaeangliae*	Humpback	COA150611Mn	CCS	Female	2015	‘Spinnaker,’ subadult, entangled in fishing gear
*Eschrichtius robustus*	Gray #1	HMSCNAU3	HMSC	Unk	Unk	Necropsy specimen from marine mammal stranding network
*Eschrichtius robustus*	Gray #2	HMSC03C2	OSU	Female	2003	Calf, entangled in fishing gear

^a^CCS = Center for Coastal Studies, Provincetown, MA; COA = Allied Whale, College of the Atlantic, Bar Harbor, ME; HMSC = Hatfield Marine Science Center, Oregon State University, Newport, OR; NAU = Northern Arizona University, Flagstaff, AZ; NBWM = New Bedford Whaling Museum, New Bedford, MA; WHOI = Woods Hole Oceanographic Institution, Woods Hole, MA.

### Baleen pulverization and hormone extraction

Once in the laboratory, plates were cleaned of surface dust by wiping three times with Kimwipes (Sigma-Aldrich, St. Louis, MO, USA) wetted with 70% isopropyl alcohol, and allowed to air-dry. A single 4 cm × 2 cm region at the base of each plate (i.e. growth zone, newest baleen) was then pulverized with a hand-held Dremel rotary tool (Model 395 Type 5) fitted with a flexible extension and a tungsten-carbide ball tip, with the drill speed set at 2. Baleen powder was collected on a weigh paper below the plate and mixed with a metal spatula. Due to the large volumes of baleen extract needed for validations, five separate 100 mg subsamples of the well-mixed baleen powder were then weighed on a digital scale to the nearest 0.1 mg, with each aliquot transferred to a 16 × 100 mm borosilicate glass tube. Considerable care was taken to avoid potential cross-contamination between specimens; all drilling occurred in a fume hood or biological safety cabinet, with comprehensive cleaning between samples (eight washes of 70% ethanol of all tools, the tungsten-carbide drilling tip, the digital scale and the work surface, with multiple changes of gloves).

Hormones were extracted by mixing each 100 mg aliquot of weighed powder with 6.00 mL of 100% high-performance liquid chromatography (HPLC)-grade methanol, vortexing 2 h, and centrifuging for 15 min at 3000 *g*. This is an adaptation of our previously published protocol (Hunt *et al.* 2014b, 2016) with the volume of methanol increased from 4.0 to 6.0 mL (per 100 mg of baleen powder) to improve extraction efficiency. Supernatant (containing hormones) was pipetted to a separate tube and dried down in a ThermoSavant SpeedVac SPD121P (Thermo Fisher Scientific, Waltham, MA, USA) under vacuum at 35°C for 5 h. Dried samples were reconstituted in 500 uL of EIA assay buffer (buffer ‘X065,’ Arbor Assays, Ann Arbor, MI, USA), sonicated 1 min, and vortexed 5 min. The 500 uL reconstitution volume was selected following initial studies in bowhead and right whale (Hunt *et al.* 2014b, 2016, 2017), in which this volume was found to concentrate samples enough for acceptable detectability of at least two hormones (progesterone and cortisol), while still retaining good assay accuracy and sufficient volume for multiple immunoassays. However, we emphasize that the ideal extraction and reconstitution protocol for baleen powder has not yet been definitively determined, and that further optimization of these methods may be possible. For each species, the five separate 500 uL extracts (originally from the same large powdered sample) were combined, vortexed 15 s to equalize hormone concentrations, and transferred to a cryovial for storage at −80°C. This is termed the ‘1:1’ (full-strength) extract and was used for all assays. The 1:1 extracts from each species were then serially diluted by progressive halving in EIA assay buffer, producing a total of eight dilutions: 1:1, 1:2, 1:4, 1:8, 1:16, 1:32, 1:64 and 1:128. All eight dilutions from all eight species were then assayed in duplicate in all eight immunoassays. Hormone assays occurred within 3 months of extraction.

In a few cases where hormone content proved to be so low or so high that only the non-linear ‘shoulder’ of the parallelism curve was recovered, additional dilutions were created that were either more dilute (e.g. 1:256, 1:512, etc.) or more concentrated (e.g. ‘2×’, twice as concentrated as 1:1), as appropriate. All dilutions were then re-assayed together in a subsequent assay, in order to recover the central linear portion of the binding curve for more accurate comparison to the slope of the standard curve.

### Hormone assays

All assays were colorimetric EIAs from the same manufacturer (Arbor Assays, Ann Arbor, MI). A single manufacturer was selected so that baleen extracts could be reconstituted into a single assay buffer that would be suitable for all assays; this avoids the need to repeatedly dry down extracts for reconstitution in separate buffers, minimizes associated pipetting losses, and hence minimizes the mg of baleen powder required from the rare specimens. The specific EIA kit manufacturer was selected based on previous successful use of certain kits (progesterone, cortisol) with bowhead and right whale baleen extract (Hunt *et al.* 2014b, 2016, 2017); additionally, several of this manufacturer’s assay kits are specifically designed for use with non-plasma sample types of wildlife. The manufacturer’s protocols were followed exactly for the progesterone, testosterone, 17β-estradiol and corticosterone EIAs. Other EIA protocols were slightly modified as follows: cortisol, standards brought up in the X065 buffer (based on technical advice from the manufacturer); aldosterone, ‘overnight’ format used but with 50ul of standards and samples; T4, ‘urine’ format used but with 50 uL of standards and samples; T3, 50uL used of standards and samples. All assays followed standard QA/QC including assay in duplicate of all non-specific binding wells, blanks (zero dose), standards and all samples, with results averaged accordingly; re-assay of any sample with coefficient of variation (CV) > 10% between wells; and exclusion of any single standard with >10% CV from the standard curve. In cases where two or more standards had CV > 10% (this was rare), the entire assay was re-run. See Table 2 for additional assay details including catalog numbers, antibody sources, manufacturer’s reported inter- and intra-assay precision (aka ‘assay variation’), sensitivity and cross-reactivities.

**Table 2:** Details of immunoassays used to test hormone detectability in baleen powder. All assays were colorimetric enzyme immunoassay kits from Arbor Assays (Ann Arbor, MI, USA). Detection limit, intra-assay precision and inter-assay precision are from manufacturer’s protocols, except for detection limit for aldosterone and T4 assays, in which due to protocol alterations the detection limit was conservatively estimated as one-half the dose of the lowest standard

Hormone (assay catalog #)	Source of antibody	Standard range (pg/mL), # standards	Detection limit (pg/mL)	Intra-assay precision^a^ (%)	Inter-assay precision^a^ (%)	Cross-reactivities
Progesterone (K025)	Mouse monoclonal	50–3200	52.9	3.9	5.7	Hydroxyprogesterones^b^: 3α, 172%; 3β, 188%; 11α, 2.7%; 11β, 147%. Pregnenolone 5.9%, other steroids <0.1%
		*n* = 7				
Testosterone (K032)	Rabbit polyclonal	41–10 000	30.6	10.9	9.3	Dihydrotestosterone 56.8%, androstenedione 0.27%, other steroids <0.1%
		*n* = 7				
17β-Estradiol (K030)	Rabbit polyclonal	39–10 000	26.5	5.1	8.4	Estrone 0.73%, other steroids <0.1%
		*n* = 5				
Cortisol (K003)	Mouse monoclonal	100–3200	45.4	8.8	8.1	Dexamethasone 18.8%, prednisolone 7.8%, corticosterone 1.2%, cortisone 1.2%, other steroids <0.1%
		*n* = 6				
Corticosterone (K014)	Sheep polyclonal	78–10 000	16.9	5.2	7.9	Desoxycorticosterone 12.3%, tetrahydrocorticosterone 0.76%, aldosterone 0.62%, cortisol 0.38%, progesterone 0.24%, dexamethasone 0.12%, other steroids <0.1%
		*n* = 8				
Aldosterone (K052)	Sheep polyclonal	3.9–4000	1.9	6.9	19.5	All tested steroids <0.1%
		*n* = 6				
Tri-iodothyronine, T3 (K056)	Sheep polyclonal	78–5000	46.6	6.3	13.4	T4, 0.88%, reverse T3 < 0.1%
		*n* = 7				
Thyroxine, T4 (K050)	Mouse monoclonal	62.5–4000	31.3	3.0	7.1	Reverse T3 89.0%, T3 5.23%
		*n* = 7				

^a^Average of three (most assays) or four (corticosterone assay) coefficients of variation reported by the assay manufacturer for various concentrations of the relevant hormone.

^b^Progesterone assay uses the ‘CL425’ antibody widely used to detect hydroxylated progesterone metabolites common in mammalian faeces (Graham *et al.*, 2001). Cross-reactivities are defined in reference to progesterone.

Parallelism was tested with assay of eight serial dilutions of baleen extract for each species, assayed alongside known-dose hormone standards. Due to microplate size limitations (12 columns, of which 2 are devoted to the standard curve), right, blue, sei, minke and fin whales were typically run in one assay, followed by bowhead, humpback, gray and a new standard curve in a separate assay, and follow-up assays for tests of additional dilutions or additional individual whales (e.g. blue #2, fin #2, gray #2). For simplicity, only the first assay’s standard curve is displayed on figures, but note that statistical testing compared each species’ data to the standard curve run in the same assay. Bowhead and right whale baleen had previously been tested for progesterone and cortisol parallelism (Hunt *et al.* 2014b, 2016, 2017); these previously published data are displayed on figures and tables, and are reanalyzed here with multiple-comparisons corrections, for ease of comparison to new data from other species.

Accuracy tests require a much greater volume of sample than parallelism tests, and therefore accuracy could not be evaluated for all hormones due to limited sample volume from these rare specimens. Thus, we prioritized accuracy testing for progesterone and testosterone, due to their importance in male and female reproductive assessment, respectively, and cortisol, due to its key role in the adrenal stress response. Cortisol was chosen for accuracy testing rather than corticosterone due to the assumption that cetaceans are ‘cortisol-dominant’, i.e. cortisol is thought to be the major circulating glucocorticoid in cetacean plasma (reviewed in St. Aubin, 2001; Atkinson *et al.*, 2015). In most cases, dilutions of 1:16 were tested for progesterone and testosterone, and 1:2 for cortisol; for a few species, stronger dilutions were tested. These dilutions were selected by consulting parallelism data for a dilution that fell between 60 and 80% percent-bound (percentage of antibody bound to labelled hormone), a region of the parallelism binding curve that typically has good mathematical accuracy while requiring a minimal volume of sample. For each accuracy test, a full standard curve, non-specific binding wells and blank (zero dose) were spiked in duplicate with an equal volume of appropriately diluted baleen extract, and the spiked standards were then assayed alongside a second standard curve that was spiked only with buffer.

### Statistical analysis

Parallelism results were graphed as percentage of antibody bound vs. log[relative dose], with the strongest dilution for each species assigned a nominal value and each subsequent dilution assigned a relative dose of 1/2 that of the previous dilution. The resulting binding curves were assessed with an *F* test for difference of slope, with the linear portion of each species’ binding curve compared to the standard curve that had been assayed in the same assay (Grotjan and Keel, 1996; Ezan and Grassi, 2000). A Bonferroni multiple-comparisons correction was used for each group of eight species tested for a certain hormone, i.e. the significance threshold for each hormone was set at *α*= 0.05/8 = 0.00625. (Note that in parallelism testing, the desired result is lack of significance, and thus Type II errors are a greater concern than Type I errors; more stringent multiple comparisons tests were not employed so as to limit risk of Type II errors.) Accuracy results were graphed as observed dose vs. known standard dose and were assessed using linear regression, with acceptable accuracy defined as *r*^2^ > 0.95 and slope within the range of 0.7–1.3 (ideal slope = 1.0) (Grotjan and Keel, 1996; Ezan and Grassi, 2000).

## Results

### Detectability and parallelism

**Figure 1:**
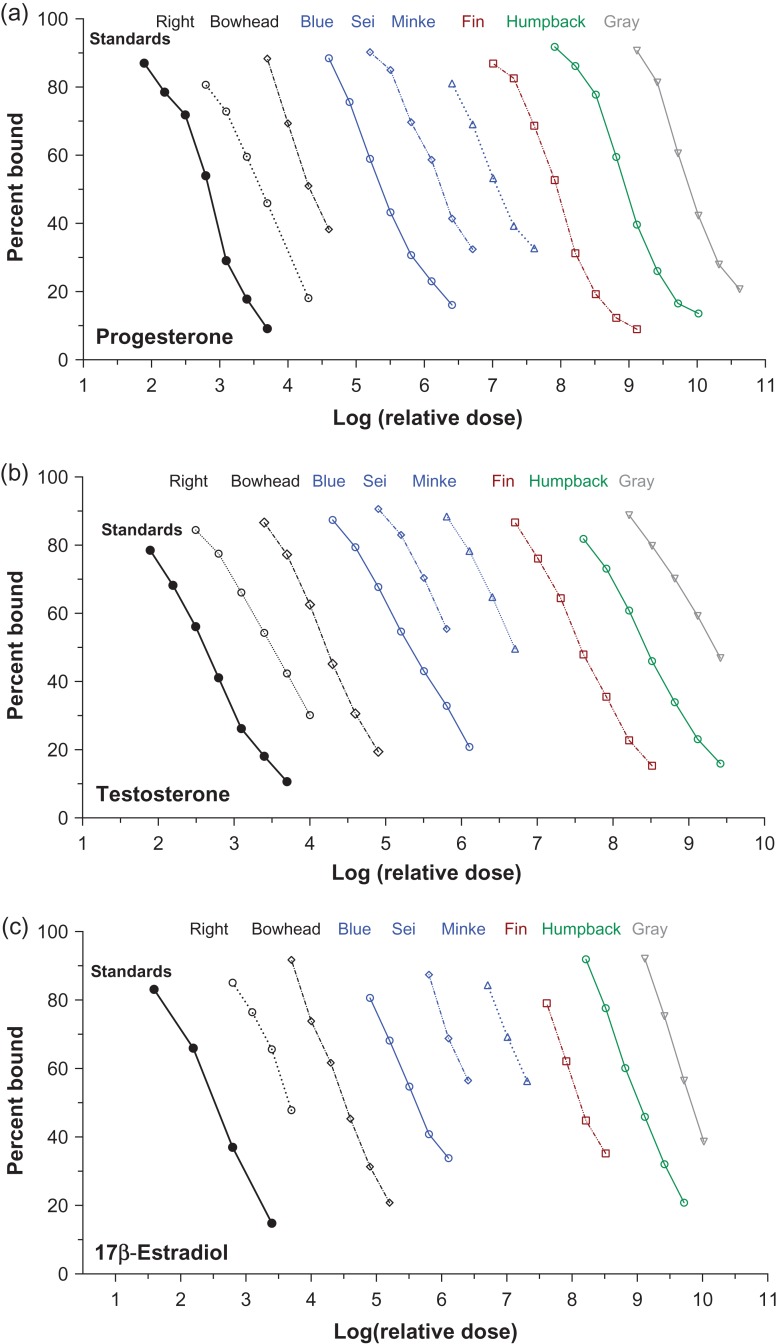
Parallelism graphs for the reproductive steroids progesterone (top), testosterone (middle) and 17β-estradiol (bottom) tested with serially diluted baleen extracts of eight whale species. Similar colours indicate closely related taxa, i.e. right (North Atlantic right whale) and bowhead are closely related, and blue, sei and minke whales are closely related (fin whale is not grouped with other *Balaenoptera* following recent phylogenetic analyses, e.g. Marx and Fordyce, 2015). Dilutions with no detectable hormone are not shown

**Figure 2:**
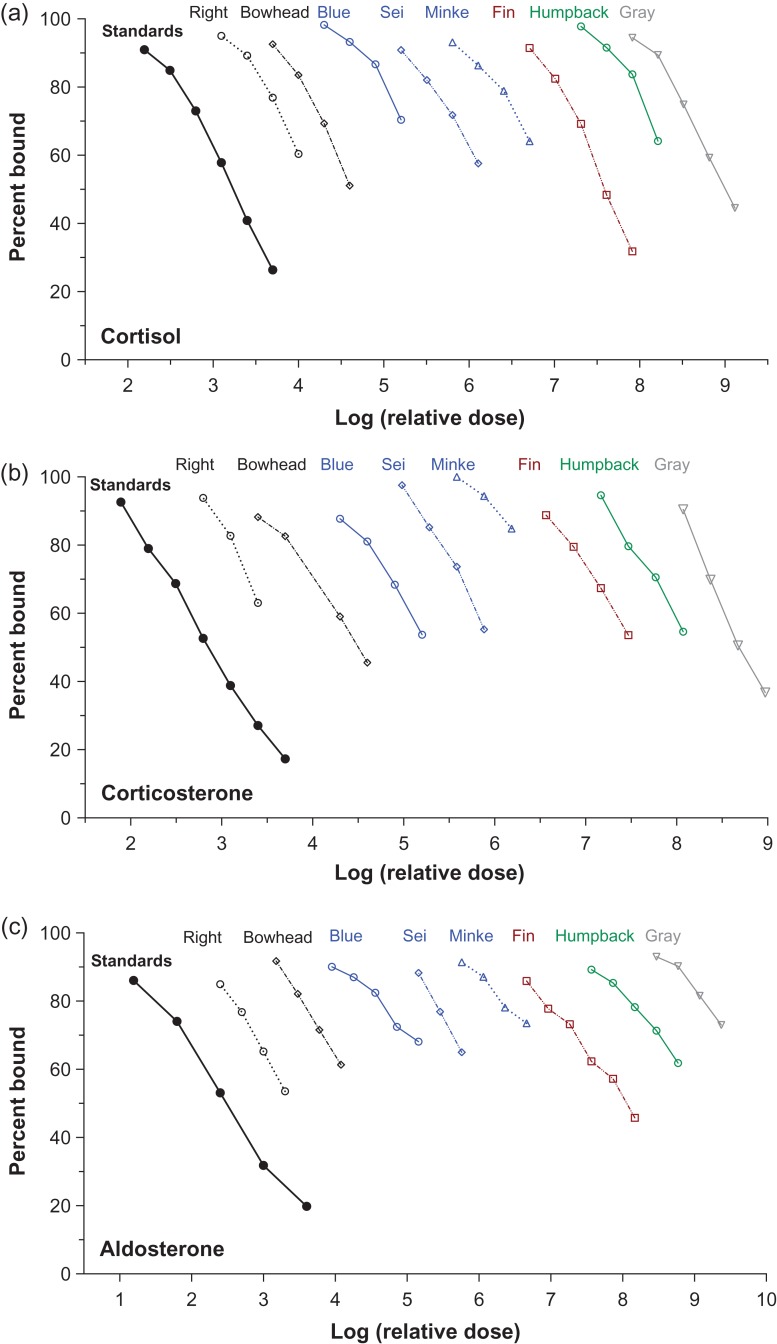
Parallelism graphs for the adrenal steroids cortisol (top), corticosterone (middle) and aldosterone (bottom) tested with serially diluted baleen extracts of eight whale species. Similar colours indicate closely related species; right = North Atlantic right whale. Dilutions with no detectable hormone are not shown

**Figure 3:**
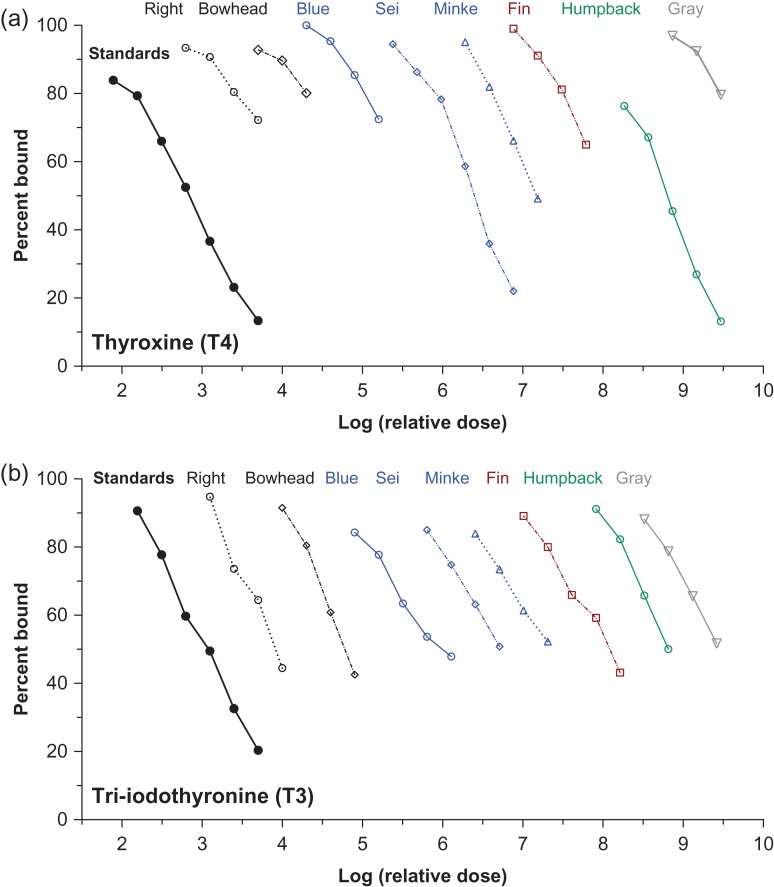
Parallelism graphs for the thyroid hormones thyroxine (T4) and tri-iodothyronine (T3) tested with serially diluted baleen extracts of eight whale species. Similar colours indicate closely related species; right = North Atlantic right whale. Dilutions with no detectable hormone are not shown

Immunoreactive hormones were detectable in baleen powder of all species tested, in all eight hormone assays. There were no significant differences in slope between the linear portions of the binding curves of serially diluted baleen extract as compared to pure hormone standards run in the same assay (Figs 1–3, Table 3; note that *x*-axes of Figs 1–3 show relative dose, not actual dose).

**Table 3:** Results of individual *F*-tests for parallelism for baleen extract of eight mysticete whales in eight enzyme immunoassays, comparing slopes of the linear portions of the standard curve and the serial dilution of baleen extract for each species. Variable degrees of freedom are due to some dilutions having undetectable hormone content for some species. All *P* values are > 0.00625, the Bonferroni-corrected alpha value for each hormone. ‘Right’ = North Atlantic right whale; T3 = tri-iodothyronine; T4 = thyroxine

Species	Progesterone	17β-Estradiol	Testosterone	Cortisol	Corticosterone	Aldosterone	T3	T4
Right^a^	*F*_*1,8*_ = 0.3856	*F*_1,5_ = 0.6831	*F*_1,9_ = 1.521	*F*_1,6_ = 3.682	*F*_1,6_ = 2.624	*F*_1,5_ = 2.051	*F*_1,7_ = 0.1184	*F*_1,6_ = 0.5985
	*P* = 0.3856	*P* = 0.4462	*P* = 0.3073	*P* = 0.1034	*P* = 0.1564	*P* = 0.2115	*P* = 0.7409	*P* = 0.4685
Bowhead^a^	*F*_1,7_ = 1.184	*F*_1,6_ = 1.898	*F*_1,10_ = 2.198	*F*_1,6_ = 0.2496	*F*_1,9_ = 2.707	*F*_1,4_ = 0.33553	*F*_1,7_ = 0.1443	*F*_1,3_ = 2.501
	*P* = 0.3126	*P* = 0.2174	*P* = 0.1690	*P* = 0.6352	*P* = 0.1343	*P* = 0.5936	*P* = 0.7153	*P* = 0.2119
Blue	*F*_1,8_ = 2.141	F_1,6_ = 1.343	*F*_1,10_ = 0.9145	*F*_1,5_ = 0.4851	*F*_1,7_ = 1.684	*F*_1,4_ = 4.317	*F*_1,7_ = 2.074	*F*_1,4_ = 5.751
	*P* = 0.1816	*P* = 0.2906	*P* = 0.3615	*P* = 0.5172	*P* = 0.2355	*P* = 0.1063	*P* = 0.1930	*P* = 0.0745
Sei	*F*_1,8_ = 1.928	*F*_1,5_ = 0.9750	*F*_1,7_ = 1.071	*F*_1,5_ = 3.718	*F*_1,6_ = 2.148	*F*_1,3_ = 1.707	*F*_1,6_ = 0.0228	*F*_1,8_ = 3.293
	*P* = 0.2024	*P* = 0.3688	*P* = 0.7530	*P* = 0.1117	*P* = 0.1931	*P* = 0.2825	*P* = 0.8849	*P* = 0.1071
Minke	*F*_1,7_ = 0.9518	*F*_1,5_ = 1.045	*F*_1,7_ = 0.2176	*F*_1,5_ = 0.7135	*F*_1,5_ = 1.630	*F*_1,3_ = 1.519	*F*_1,6_ = 0.3154	*F*_1,5_ = 0.5093
	*P* = 0.3618	*P* = 0.3536	*P* = 0.6551	*P* = 0.4368	*P* = 0.2577	*P* = 0.3055	*P* = 0.5947	*P* = 0.1709
Fin	*F*_1,10_ = 1.186	*F*_1,5_ = 3.962	*P*_1,10_ = 0.0488	*F*_1,6_ = 5.015	*F*_1,7_ = 1.607	*F*_1,7_ = 1.575	*F*_1,7_ = 0.0835	*F*_1,4_ = 2.158
	*P* = 0.3016	*P* = 0.1032	*P* = 0.8296	*P* = 0.0664	*P* = 0.2455	*P* = 0.2497	*P* = 0.7809	*P* = 0.2157
Humpback	*F*_1,10_ = 1.210	*F*_1,6_ = 2.826	*F*_1,10_ = 0.8866	*F*_1,6_ = 5.345	*F*_1,7_ = 0.0069	*F*_1,4_ = 2.005	*F*_1,7_ = 0.8685	*F*_1,5_ = 0.1192
	*P* = 0.2970	*P* = 0.1438	*P* = 0.3686	*P* = 0.0601	*P* = 0.9359	*P* = 0.2297	*P* = 0.3824	*P* = 0.7440
Gray	*F*_1,8_ = 0.0118	*F*_1,4_ = 20.3	*F*_1,7_ = 0.2623	*F*_1,7_ = 0.2437	*F*_1,8_ = 2.726	*F*_1,3_ = 0.1849	*F*_1,6_ = 0.1006	*F*_1,6_ = 2.089
	*P* = 0.9163	*P* = 0.0108	*P* = 0.6243	*P* = 0.6366	*P* = 0.1373	*P* = 0.6962	*P* = 0.7619	*P* = 0.1985

^a^Right and bowhead results for progesterone and cortisol are from data previously published in Hunt *et al.* (2014b, 2016) and are presented here for comparison.

In some cases hormone concentration was quite low, such that only the uppermost part of the binding curve was recovered (i.e. sometimes only two or three of the most concentrated dilutions had detectable hormone), particularly for cortisol, aldosterone and T4. In most such cases parallelism was still testable, but cortisol especially tended to have low concentration in baleen extract and initially showed imperfect parallelism for blue whale #1, fin whale #1 and gray whale #1 (data not shown). These three individual whales had extremely low baleen cortisol concentrations and thus it is likely that only the non-linear ‘shoulder’ portion of the upper part of the binding curve was recovered. Upon testing baleen from a second whale for these three species (blue whale #2, fin whale #2 and gray whale #2), in all three cases the second whale had higher baleen cortisol content, the linear portion of the curve was recovered, and parallelism was good (these three individuals’ data are shown in Fig. 2 and in the cortisol column in Table 3).

Assay accuracy was acceptable for all species for the three assays tested (progesterone, testosterone and cortisol), with linear relationships of observed vs. expected concentration and a slope within the desired range of 0.7–1.3 (Table 4). The progesterone assay tended to have slightly ‘shallow’ accuracy, i.e. slope of ~0.8 rather than the ideal of 1.0, but accuracy was nonetheless acceptable (straight line that consistently distinguished low from high concentrations).

**Table 4:** Results of accuracy tests (‘matrix effect’ tests) for progesterone, testosterone and cortisol assays tested with baleen extract of eight mysticete whale species. Slope of observed dose in standards spiked with baleen extracted vs. unspiked standards is shown, along with coefficient of determination (*r*^2^) of the linear regression line. A slope close to 1.0 (acceptable range = 0.7–1.3) and *r*^2^ close to 1.00 indicates that the assay can quantify hormone in the presence of baleen extract with acceptable mathematical accuracy. Each assay was tested with the dilution shown in parentheses (where 1:1 = extract produced from 100 mg baleen powder vortexed with 6.0 mL methanol, dried, and brought up in 0.5 mL assay buffer). ‘Right’ = North Atlantic right whale.

	Progesterone	Testosterone	Cortisol
Right^a^	Slope = 0.7360	Slope = 0.9268	Slope = 1.060
	*r*^2^ = 0.9968	*r*^2^ = 0.9999	*r*^2^ = 0.9906
	(1:4)	(1:16)	(1:1)
Bowhead^a^	Slope = 0.7194	Slope = 1.046	Slope = 1.013
	*r*^2^ = 0.9989	*r*^2^ = 0.9942	*r*^2^ = 0.9998
	(1:4)	(1:16)	(1:1)
Blue	Slope = 0.8969	Slope = 1.067	Slope = 1.036
	*r*^2^ = 0.9975	*r*^2^ = 0.9963	*r*^2^ = 0.9999
	(1:16)	(1:16)	(1:4)
Sei	Slope = 0.7953	Slope = 1.017	Slope = 1.025
	*r*^2^ = 0.9860	*r*^2^ = 0.9998	*r*^2^ = 0.9997
	(1:16)	(1:16)	(1:4)
Minke	Slope = 0.8534	Slope = 1.017	Slope = 1.010
	*r*^2^ = 0.9991	*r*^2^ = 0.9939	*r*^2^ = 0.9928
	(1:16)	(1:16)	(1:4)
Fin	Slope = 0.7927	Slope = 1.026	Slope = 1.037
	*r*^2^ = 0.9854	*r*^2^ = 0.9980	*r*^2^ = 0.9993
	(1:16)	(1:16)	(1:4)
Humpback	Slope = 0.8819	Slope = 1.122	Slope = 1.121
	*r*^2^ = 0.9991	*r*^2^ = 0.9995	*r*^2^ = 0.9988
	(1:16)	(1:16)	(1:4)
Gray	Slope = 0.9280	Slope = 0.9241	Slope = 0.968
	*r*^2^ = 0.9986	*r*^2^ = 0.9999	*r*^2^ = 0.9984
	(1:16)	(1:4)	(1:2)

^a^Right and bowhead results for progesterone and cortisol are from data previously published in Hunt *et al.* (2014b, 2016) and are presented here for comparison.

Certain hormones exhibited differences in relative concentration that appeared consistent across species. Corticosterone:cortisol ratio was > 1.0 (i.e. more corticosterone than cortisol) for all individuals tested except the minke whale. Corticosterone concentration was occasionally as much as two times higher (gray whale) or three times higher (bowhead whale) than cortisol. Among the reproductive hormones, estradiol tended to be present at lower concentration than either progesterone or testosterone. For thyroid hormones, T3 concentrations were similar across all individual whales (across species) but T4 was highly variable, with high concentrations in the humpback and sei whale baleen, and low concentrations in other individuals.

## Discussion

Immunoreactive steroid and thyroid hormones were easily detectable in baleen extracts from eight mysticete whale species tested with commercially available immunoassay kits, and parallelism and accuracy results were consistently good. The validation results indicate that the specific EIAs tested here perform well with baleen extract, with good binding affinity (as indicated by good parallelism) and accurate discrimination of high from low concentrations despite potential matrix effects (i.e. due to baleen extract). The good validation results are notable given that baleen extracts were not filtered and not purified via column chromatography or other means. In fact, baleen extracts at the stronger dilutions of 1:1, 1:2 and 1:4 were visibly cloudy, with the 1:1 ‘full-strength’ extract often being nearly opaque, yet parallelism results indicate that the assays performed well even at these stronger concentrations. It is striking that parallelism was so consistently good across 64 separate parallelism tests; our interpretation is that all steroid and thyroid hormones are routinely deposited in whale baleen. Even so, we recommend that future studies employ HPLC, mass spectrometry or other methods to verify the chemical identity of immunoreactive substances that the EIA antibodies are detecting.

Generally, the assay sensitivity and hormone concentrations that we observed indicate that it should be feasible to use EIAs to assay for a full panel of hormones from a relatively small mass of baleen powder. Most EIAs require 100 uL of sample for testing in duplicate (e.g. 50 uL per well × 2 wells), but diluted extract can be used for certain assays (particularly the reproductive steroids). Our present extraction method generates 500 uL of extract from a 100 mg powdered baleen sample. If the hormone concentrations seen in these individual whales are typical, we calculate it would be possible to assay all eight hormones with a total of ~250 uL of such extract. This compares favourably to volumes needed for mass spectrometry, which can detect a full panel of steroids in a single run but often requires a larger total volume of sample. Continued optimization of extraction protocol along with testing of additional assay dilutions may enable further reduction of the mass of baleen powder needed from rare specimens.

Of the eight hormones, progesterone and testosterone were consistently present at highest concentration and were easily detectable in baleen extracts of all species studied. Estradiol was easily detectable in all samples as well, though at slightly lower concentrations. The adrenal steroids and thyroid hormones were generally present at lower concentrations than the reproductive steroids and in some cases were barely detectable (particularly cortisol, aldosterone and T4). All of these patterns are consistent with relative concentrations typically seen in mammalian plasma (K. Hunt, pers. obs.); estradiol tends to circulate at lower concentration than progestogens and androgens, and adrenal and thyroid hormones also are often low in plasma and/or in peripheral tissues (e.g., T4, is converted to T3 by peripheral tissues, resulting in low concentrations of T4 in many tissues; Bentley, 1998).

In most individual whales, baleen corticosterone was markedly higher in concentration than baleen cortisol, sometimes 2-fold or 3-fold higher. Similar patterns have recently been reported in right whale baleen, in which baleen corticosterone concentrations are routinely ~4× higher than baleen cortisol (Hunt *et al.*, 2017). Historically, cortisol has been assumed to be the more abundant of the two glucocorticoids in mysticete plasma (reviewed in St Aubin, 2001; Atkinson *et al.*, 2015). However, plasma glucocorticoids have only been assessed in mysticete whales in a few cases, all involving acute stress situations (stranding or hunting; e.g. Kjeld, 2001, Rolland et al., 2017). It is therefore unclear which of the two glucocorticoids may normally be more abundant in plasma and, importantly, which of the two may be more useful to study for questions regarding stress physiology. From a sample-mass perspective, assay of the more abundant glucocorticoid—corticosterone, it seems—would require a smaller mass of baleen powder, since the extract could be routinely diluted for assay, as described above. This issue is of some importance given high interest in assessing stress of historic populations from which only small subsamples may be available (e.g. small amounts of baleen powder left over from stable-isotope analyses). However, since there are some indications that cortisol and corticosterone may respond differently to acute vs. chronic stress in mammals (Koren *et al.*, 2012), it may be most informative to study both glucocorticoids where possible.

In sum, our findings indicate that commercially available immunoassays appear to be a viable technique for analysis of hormone concentrations in baleen extracts from a wide variety of mysticete whale species. We caution that our results represent very small sample sizes, in most cases single samples from single individuals. However, initial assay validations of parallelism and accuracy usually are generalizable within a given species as long as the same extraction method and same assay kit is used, i.e. with the same antibody. Our results may only apply to the particular extraction method used here (methanol extraction, with reconstitution into appropriate assay buffer), and may also be limited to the specific commercial assay kits tested. Previous experience has shown that different assay kits, even if marketed as detecting the same hormone, typically employ different antibodies that may have radically differing cross-reactivities to other hormones and may also exhibit idiosyncratic reactions to non-plasma sample matrices (K. Hunt, pers. obs.). We therefore advise that validation tests be repeated if different commercial EIA kits are used or if extraction protocol is altered.

Overall, the good performance of commercial immunoassay kits for multiple steroid and thyroid hormones across a wide range of mysticete taxa bodes well for the potential utility of the baleen-hormone technique for investigating large whale physiology for conservation and management purposes, particularly given available historic archives of baleen. The range of hormones in baleen includes the major reproductive steroids of both males and females, two major adrenal steroids (cortisol, corticosterone) that are intimately involved in the mammalian stress response, a mineralocorticoid (aldosterone) involved both in stress responses and in osmotic regulation, and the two major thyroid hormones. Baleen hormone analysis therefore may have the potential to enable retrospective analysis of multi-year patterns of whale physiology that have until now been very difficult to address, potentially including pregnancy and inter-calving intervals, estrous cycles of females, testosterone cycles of males, age of sexual maturity in both sexes, metabolic rate and long-term responses to acute and chronic stress. We emphasize that many validations remain to be performed, such as mass spectrometry and HPLC to verify hormone chemical identity, stable-isotope studies to assess variability in baleen growth rate within and across species as well as across seasons, tests of variability of hormone deposition within and across baleen plates, further optimization of extraction protocols, and, importantly, physiological validations—verification that patterns in baleen hormones down the length of full baleen plates correlate with known physiological histories of individual whales. Once these further tests are performed, baleen hormone analyses may be a valuable new addition to the available toolkit for studies of physiology in the large whales.
